# Clear but Not Simple: Diagnostic Challenges of a Rare Case of Hyalinising Clear Cell Carcinoma of the Palate

**DOI:** 10.7759/cureus.93774

**Published:** 2025-10-03

**Authors:** Radhika Ramgundamwar, Vaishali Narayen, Shyam NDVN, Paremala Konda, Kiran Kumar Gundamaraju

**Affiliations:** 1 Department of Oral and Maxillofacial Pathology, Government Dental College and Hospital, Hyderabad, IND

**Keywords:** cytokeratin 8/18, hyalinising clear cell carcinoma, immunohistochemistry, mucicarmine, periodic acid schiff, salivary gland carcinoma, subtotal maxillectomy

## Abstract

Hyalinising clear cell carcinoma is an uncommon neoplasm arising in minor salivary glands. It is a rare low-grade malignant tumour composed of clear cytoplasmic cells. This case report outlines the presentation of a 36-year-old female with a history of growth in the palatal region for six months. Examination revealed a mass originating from the left hard palatal region, and on correlation with radiographic examination, biopsy was advised. Pathological examination revealed typical clear cells arranged in anastomosing trabeculae, cords, nests, or solid sheets with hyalinising stroma. These cells were strongly positive for periodic acid-Schiff (PAS) but were negative for mucicarmine. Immunohistochemically, these neoplastic cells were immunoreactive to cytokeratin 8/18 but negative to smooth muscle actin, which aids in the diagnosis. Left subtotal maxillectomy was done for the excision of the tumour, followed by radiotherapy, along with follow-up of 18 months without recurrence.

## Introduction

Hyalinising clear cell carcinoma (HCCC) is a rare malignant neoplasm of the salivary glands, first described by Milchgrub et al. in 1994 [1]. It accounts for approximately 1% of all salivary gland tumours and typically arises in minor salivary glands, most commonly in the hard and soft palate [1,2]. Histologically, HCCC is characterised by nests of clear cells embedded within a densely hyalinized stroma. The clear cell appearance is attributed to the abundant presence of intracellular glycogen, which can be confirmed by periodic acid-Schiff (PAS) staining and diastase sensitivity [3].Despite its malignant nature, HCCC generally presents as a slow-growing, painless mass and is associated with a low risk of recurrence.

The microscopic features of HCCC often overlap with other salivary gland neoplasms, posing significant diagnostic challenges.

Herein, we report a rare case of HCCC located in the left hard palate. A review of the literature is also presented to underscore the diagnostic complexities and considerations in the management of this uncommon entity.

## Case presentation

**Figure 1:**
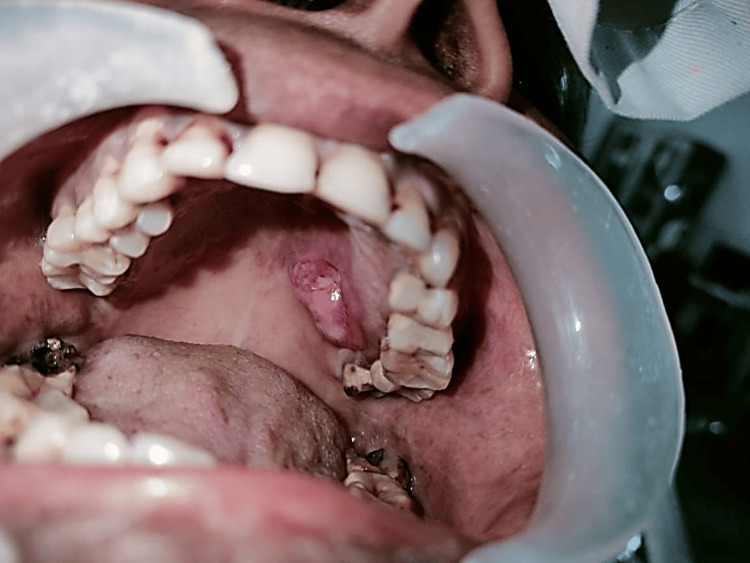
Clinical picture showing proliferative growth on the left hard palate region.

A 36-year-old female with no relevant medical, dental, or family history presented with a complaint of a growth on the left palatal region for the past six months. On head and neck examination and intra-oral examination, a solitary, firm, non-tender, 2 x 2 cm^2^ ulcero-proliferative growth on the left hard palatal region, above the mucosal surface, was appreciable. On palpation, the swelling was non-tender and indurated (Figure 1).

**Figure 2:**
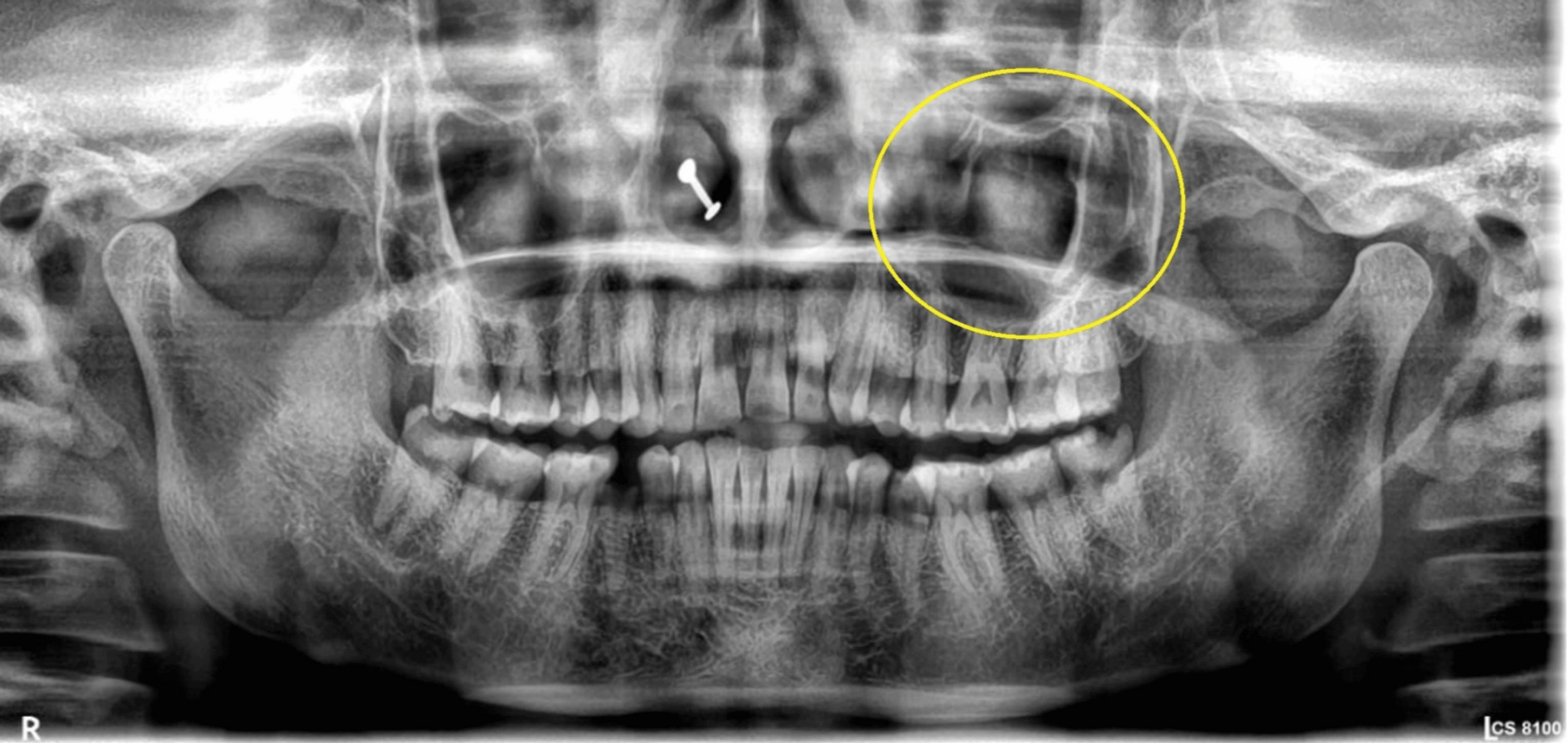
Panoramic radiograph revealed well-defined radiolucency with radiopacity in the left hard palatal region.

On radiographic examination, an ill-defined, circular, unilocular radiolucency with radiopacity was seen in the left hard palatal region at the 24-27 tooth region (Figure 2).

On the basis of the above clinical and radiographic findings, a provisional diagnosis of solitary salivary gland tumour in relation to the left hard palate was given. Incisional biopsy from the site of the left hard palatal region was done.

**Figure 3:**
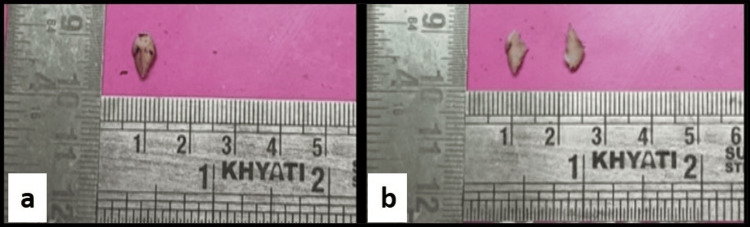
(a) A single creamish-white soft tissue bit measuring about 1.4 x 0.5 cm2, firm in consistency. (b) The soft tissue bit was cut into two pieces.

On gross examination, a single creamish-white soft tissue bit was received, measuring about 1.4 x 0.5 cm^2^, which was firm in consistency. The tissue bit was cut into two pieces (Figure 3).

**Figure 4:**
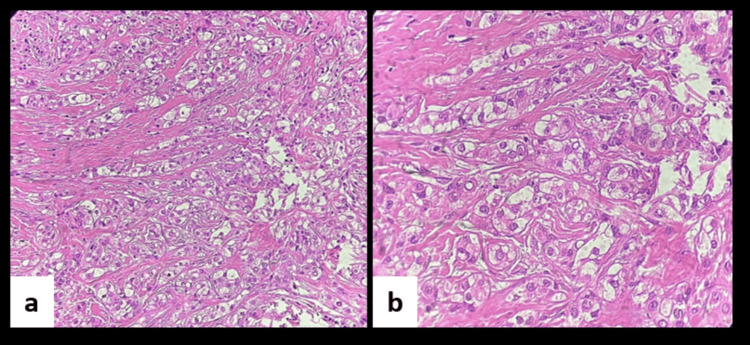
(a) Photomicrograph showing clear cells in hyalinising stroma (H&E, 20x). (b) Photomicrograph in higher magnification showing round to polygonal cells with centrally placed hyperchromatic nuclei and clear cytoplasm (H&E, 40x). H&E: hematoxylin & eosin.

Microscopic evaluation of soft tissue sections demonstrated a fibrocellular connective tissue stroma containing tumour cells organised in cords and nests, composed predominantly of clear cells, and encased within prominent zones of stromal hyalinization. These clear cells appeared round to polygonal with centrally placed hyperchromatic nuclei and clear cytoplasm. At some foci, cells showed plump nuclei with granular eosinophilic cytoplasm. Focal areas showing surface epithelium were also evident (Figure 4).

**Figure 5:**
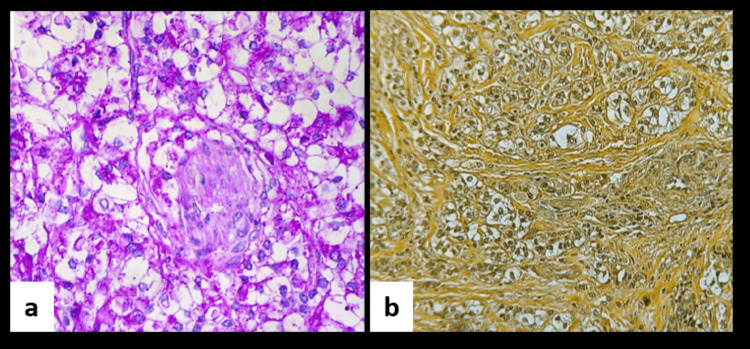
Photomicrograph showing positive staining for periodic acid-Schiff (PAS) stain (PAS stain, 40x). (b) Photomicrograph showing negative staining for mucicarmine (mucicarmine stain, 40x).

To elucidate the nature of the clear cells, two special stains were employed. The majority of tumour cells exhibited a round to polygonal morphology with cytoplasmic positivity for PAS, while mucicarmine staining was negative (Figure 5). The PAS positivity indicated intracellular glycogen accumulation as the underlying cause of the clear cell appearance, and the absence of mucicarmine staining effectively excluded mucin production.

**Figure 6:**
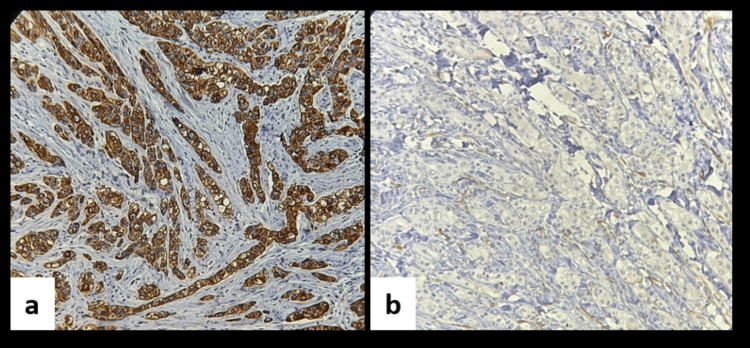
(a) Immunohistochemistry showing clear cells with strong positivity for cytokeratin 8/18 (20x). (b) Immunohistochemistry showing clear cells with negativity for smooth muscle actin (20x).

Immunohistochemical analysis was performed to determine the lineage of the clear cells. Tumour cells demonstrated positivity for cytokeratin 8/18, supporting a glandular epithelial origin. Conversely, the absence of smooth muscle actin expression excluded a myoepithelial phenotype. These findings effectively ruled out differential diagnoses such as the clear cell variant of mucoepidermoid carcinoma and clear cell myoepithelioma (Figure 6).

**Figure 7:**
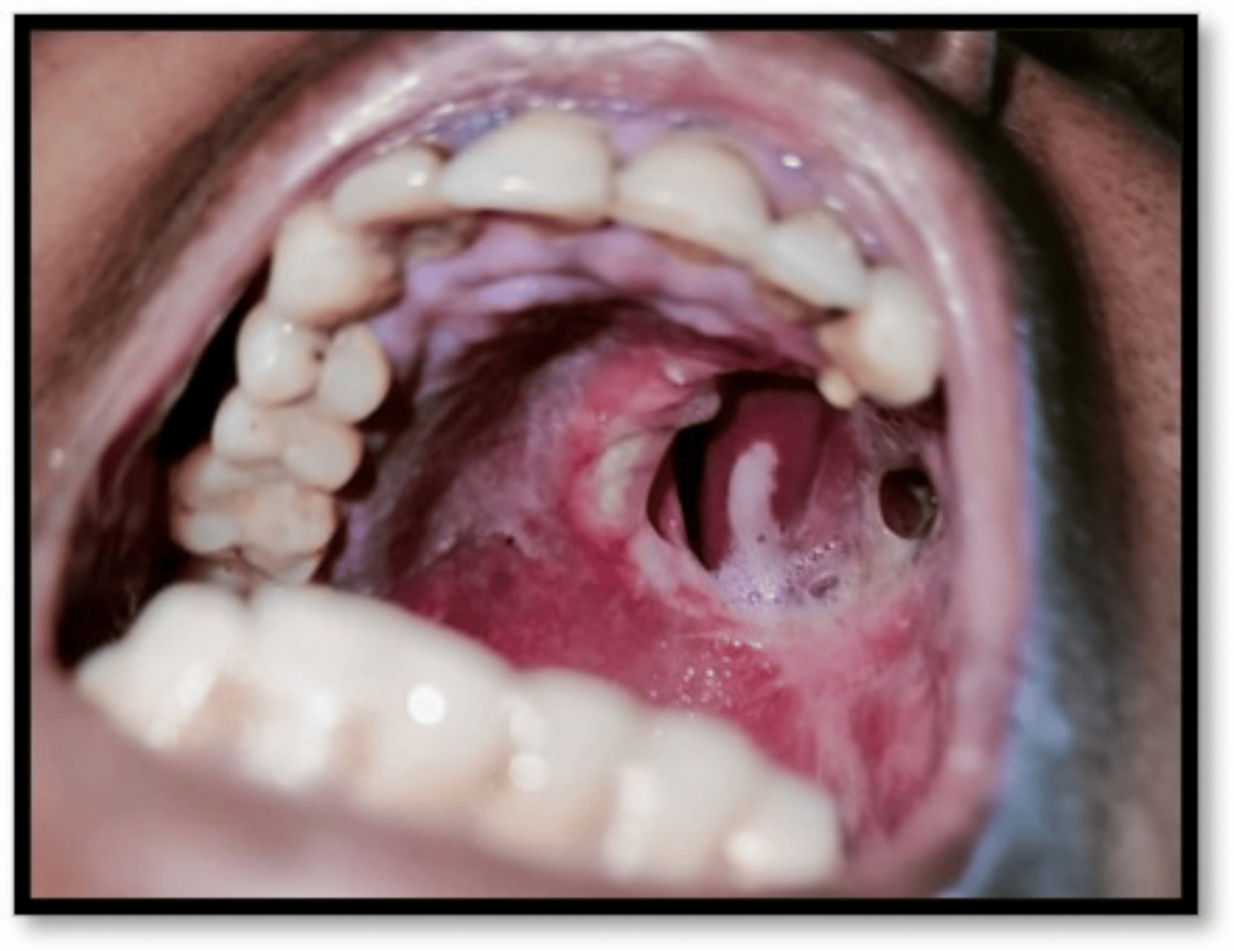
Postoperative image after excision of the tumour with left subtotal maxillectomy.

Based on the cumulative histopathological, special stains, and immunohistochemical findings, a final diagnosis of hyalinising clear cell carcinoma was established. The patient underwent a planned left subtotal maxillectomy (Figure 7), followed by adjuvant radiotherapy totalling 60 Gy in 30 fractions for two months. Postoperative prosthetic rehabilitation by a custom-made obturator was successfully completed, and the patient remains disease-free with no significant treatment-related adverse effects at 18 months of follow-up.

## Discussion

HCCC of the salivary glands is a rare malignant neoplasm that gained recognition following its initial description by Milchgrub et al. in 1994 [1,2].Since then, reporting of this entity has increased significantly. Literature suggests that HCCC occurs almost exclusively in the intraoral minor salivary glands, with a prevalence ranging from 0.2% to 1%, most commonly affecting the palate [3]. Epidemiologically, females are affected approximately twice as often as males, with elderly women being the predominant demographic. Clinically, HCCC presents as an indolent, painless, submucosal mass. Microscopically, HCCC is characterised by tumour cells with optically clear cytoplasm, well-defined borders, and centrally located nuclei. These cells exhibit minimal nuclear pleomorphism and infiltrative growth patterns [4-6].

Special stains play a crucial role in the diagnostic evaluation of clear cell lesions, particularly in distinguishing the biochemical nature of the cytoplasmic clearing. In salivary gland pathology, clear cells may contain glycogen, mucin, lipid, or other substances, each requiring specific staining techniques for accurate identification. PAS stain is commonly used to detect glycogen, which appears magenta and is typically diastase-sensitive, supporting diagnoses such as HCCC. Mucicarmine stain, on the other hand, highlights intracellular mucin, aiding in the identification of mucin-producing tumours like the clear cell variant of mucoepidermoid carcinoma. The strategic use of these stains, in conjunction with immunohistochemistry, enhances diagnostic specificity and helps differentiate morphologically similar entities with overlapping clear cell features [1].

Immunohistochemical evaluation plays a critical role in distinguishing HCCC from other clear cell neoplasms. However, the sensitivity and specificity of these markers are sometimes insufficient for definitive diagnosis, posing a challenge for specialists. HCCC of the salivary glands typically expresses epithelial markers such as cytokeratin, epithelial membrane antigen (EMA), and carcinoembryonic antigen (CEA), but lacks expression of myoepithelial markers, including S-100 protein, actin, vimentin, smooth muscle actin, and glial fibrillary acidic protein (GFAP). In this case, cytokeratin 8/18 helps in confirming the glandular origin of clear cells. The negativity for smooth muscle actin aids in ruling out the myoepithelial origin [5]. Recent studies have consistently identified the Ewing sarcoma breakpoint region 1-activating transcription factor 1 (EWSR1-ATF1) fusion gene in HCCC, while EWSR1 rearrangements have not been detected in other salivary gland tumours, highlighting the specificity of this genetic alteration to HCCC [7].

The treatment of choice for HCCC cases is wide surgical excision with adjuvant radiotherapy. It has a low rate of recurrence, being 17%, and the metastatic rate is 21%. Close follow-up, like 18 months in the present case, is, therefore, important in HCCC of the palate. A total of 11 out of 52 cases of HCCC occurring in the palate are documented in the literature [8,9].

## Conclusions

HCCC is a low-grade salivary gland neoplasm with the characteristic presence of clear cells in hyalinised stroma. Clear cell neoplasms mimicking this tumour can be excluded by special stains and immunohistochemistry techniques, which help in achieving a precise diagnosis. Minimally invasive surgical treatment should be considered, given the characteristics of HCCC, which differ from those of squamous cell carcinoma.
